# Unilateral Carpal Tunnel Syndrome Caused by an Occult Ganglion in the Carpal Tunnel: A Report of Two Cases

**DOI:** 10.1155/2014/589021

**Published:** 2014-07-06

**Authors:** Merter Yalcinkaya, Yunus Emre Akman, A. Erdem Bagatur

**Affiliations:** ^1^Department of Orthopaedic Surgery and Traumatology, Metin Sabanci Baltalimani Bone Diseases Training and Research Hospital, Rumeli Hisari Cad. No. 62, Baltalimani, Sariyer, 34470 Istanbul, Turkey; ^2^Department of Orthopaedic Surgery and Traumatology, Medicana International Istanbul Hospital, Beylikdüzü Cad. No. 3, Beylikdüzü, 34520 Istanbul, Turkey

## Abstract

Carpal tunnel syndrome (CTS) usually presents bilaterally and a secondary nature should be suspected in patients with unilateral symptoms, especially those with a long-standing history, and when the symptomatic hand shows severe neurophysiologic impairment, while the contralateral hand is neurophysiologically intact. Space-occupying lesions are known to cause CTS and the incidence of space-occupying lesions in unilateral CTS is higher than that of bilateral CTS. It is easy to detect a mass when it is palpable; however, occult lesions are usually overlooked. Whenever a patient presents with unilateral symptoms and unilateral neurophysiologic impairment, the possibility of a space-occupying lesion compressing the median nerve should be kept in mind in the differential diagnosis. This study presents two cases with an occult ganglion in the carpal tunnel compressing the median nerve and causing unilateral symptoms of CTS. We stress on the importance of imaging studies in patients with unilateral symptoms that are usually not used in CTS. The reported patients were evaluated and magnetic resonance images revealed an intratunnel space-occupying lesion.

## 1. Introduction

Any process that may lead to a rise in pressure in the carpal tunnel may cause median nerve compression and carpal tunnel syndrome (CTS). Since CTS presents bilaterally in many cases [1–3], the probability of a certain etiology should be considered when the condition is unilateral. Space-occupying lesions are among the etiologic factors that may cause CTS, and the incidence of space-occupying lesions in unilateral CTS is higher than that of bilateral CTS [4]. A secondary nature should be suspected in patients with unilateral symptoms, especially those with a long history, and when the symptomatic hand shows severe neurophysiological impairment but the contralateral hand is neurophysiologically intact. Imaging studies may be obtained in patients in whom the possibility of having idiopathic CTS is unlikely to occur. This study presents two patients with CTS secondary to a ganglion in the carpal tunnel, causing unilateral median nerve compression.

## 2. Case Reports

### 2.1. Case  1

A 31-year-old woman, a school teacher, presented with numbness, loss of strength, and awakening pain in the right hand of 1 year's duration. On clinical examination a mild thenar atrophy was observed, and Phalen's test and Tinel's sign were positive.

Nerve-conduction studies of the median nerve revealed a distal sensory latency (DSL) of 5.3 ms, a distal motor latency (DML) of 5.9 ms, a sensory nerve-conduction velocity (SNCV) of 38 m/s, and a motor nerve amplitude (MA) of 3.1 mV in the right side. Pathological needle electromyography (EMG) findings were also present. The left hand was neurophysiologically intact.

A DSL of more than 3 ms, an abductor pollicis brevis muscle-to-wrist DML of more than 4 ms, an index finger-to-wrist SNCV of 50 m/s, and the lowest limit of an amplitude of 4 mV were considered normal. Pathological EMG findings included fibrillation activity, reduced recruitment, and abnormalities in the configuration of the motor unit action potential. CTS was classified according to neurophysiological test results as mild (prolonged DSL), moderate (abnormal DSL and prolongation of DML), or severe (prolonged DSL and DML with an absence of sensory nerve action potential, low MA, or absent thenar compound muscle action potential, and findings compatible with axonal injury in EMG) [5].

**Figure 1:**
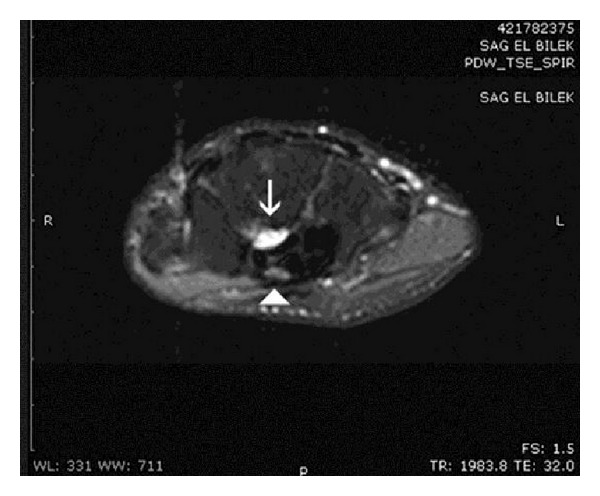
MRI revealed a space-occupying lesion (arrow) in the carpal tunnel and the median nerve (arrowhead) with high signal intensity on fat-suppressed proton density axial images of the wrist.

**Figure 2:**
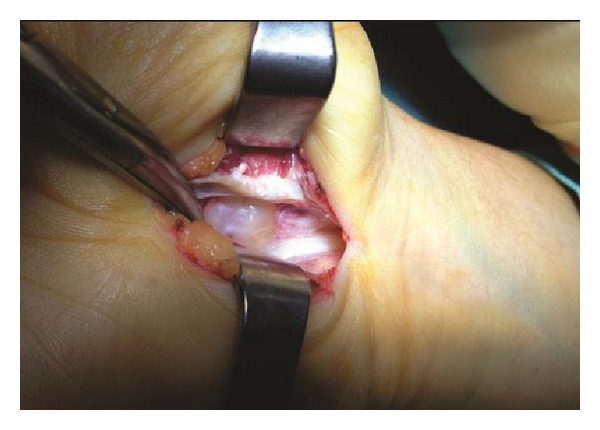
Intraoperative photograph shows the ganglion in the carpal tunnel. The flexor tendons and the median nerve were retracted.

Both clinical symptoms and signs and the neurophysiological tests showed severe [5] CTS in one hand while the contralateral hand was completely healthy, implying a secondary disease. No external signs or palpable masses were present. Magnetic resonance imaging (MRI) showed a 12 × 10 × 6 mm cystic space-occupying lesion with well-defined margins resembling a ganglion, originating from the posterior wall of the carpal tunnel and compressing the flexor tendons. MRI also showed flattening of the median nerve at the hamate level, palmar bowing of the flexor retinaculum, and increased signal intensity of the median nerve (Figure 1). Open carpal tunnel release and mass excision were performed through a palmar incision (Figure 2). Histological examination revealed thin connective tissue capsule made up of compressed collagen fibers lined with flattened cells without a synovial or epithelial lining, consistent with a ganglion. The patient reported relief of all symptoms postoperatively, and no recurrence had occurred at 1-year follow-up.

### 2.2. Case  2

A 27-year-old woman, a housewife, presented with numbness and awakening pain in the right hand of 8 month's duration. On clinical examination hypoesthesia on palmar side of the index and middle fingers was present and Phalen's test and Tinel's sign were positive.

Nerve-conduction studies of the median nerve revealed a DSL of 4.9 ms, a DML of 5.4 ms, a SNCV of 40 m/s, and a motor nerve amplitude (MA) of 3.3 mV in the right side. Pathological EMG findings were also present. The left hand was neurophysiologically intact.

**Figure 3:**
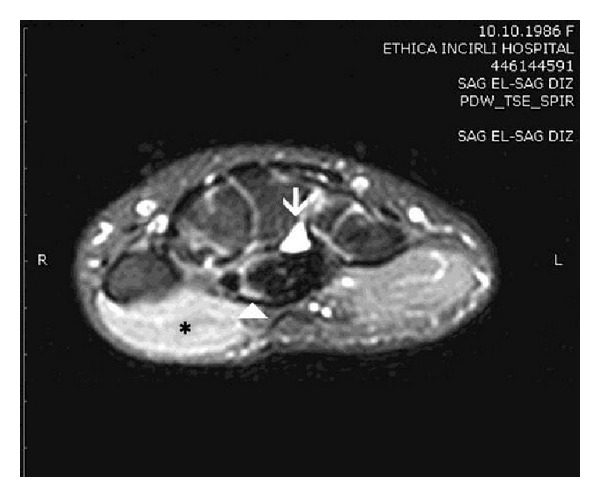
MRI revealed a space-occupying lesion (arrow) in the carpal tunnel and the median nerve (arrowhead) with high signal intensity and high signal intensity in the thenar muscles consistent with acute denervation (asterix) on fat-suppressed proton density axial images of the wrist.

**Figure 4:**
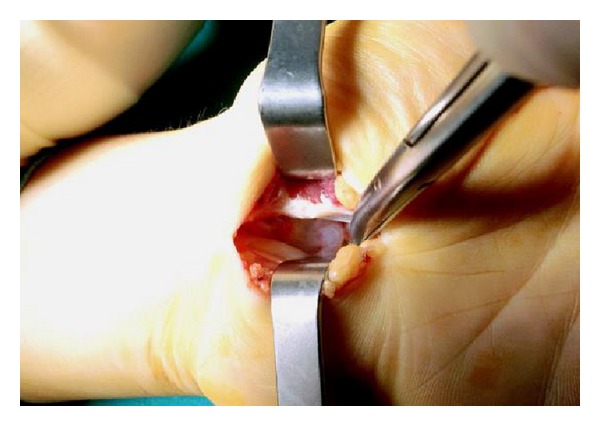
Intraoperative photograph shows the ganglion in the carpal tunnel.

Both clinical symptoms and signs and the neurophysiological tests showed severe [5] CTS in one hand while the contralateral hand was completely healthy, implying a secondary disease. MRI showed a 10 × 9 × 6 mm cystic space-occupying lesion with well-defined margins resembling a ganglion, originating from the posterior wall of the carpal tunnel, and flattening of the median nerve at the hamate level, palmar bowing of the flexor retinaculum, increased signal intensity of the median nerve, and acute denervation of the thenar muscles (Figure 3). Open carpal tunnel release and mass excision were performed through a palmar incision (Figure 4). Histological examination revealed findings consistent with a ganglion. The patient reported relief of all symptoms postoperatively, and no recurrence had occurred at 14-month follow-up.

## 3. Discussion

Although CTS is usually idiopathic, a certain etiology can be detected in some patients. Space-occupying lesions, tenosynovitis, gouty tophus, vascular anomalies, or malunited distal radial fractures may lead to CTS.

Although imaging studies for the diagnosis of CTS are becoming increasingly popular, most surgeons condemn these expensive studies as unnecessary. However, some authors report that ultrasound is an accurate and useful diagnostic tool in patients with CTS, with a sensitivity of 99% and specificity of 100%, that can be used as the initial diagnostic test in patients presenting with clinical symptoms of CTS, because it is equivalent to neurophysiological studies and provides additional valuable anatomical information [6].

We prefer using neurophysiological tests instead of imaging studies for the diagnosis of CTS when a diagnostic tool is necessary. However, imaging provides additional information compared with that obtained from clinical tests and neurophysiological studies; by allowing direct visualization of the compressed median nerve and the carpal tunnel content, imaging studies can reveal the causes of secondary CTS, depicting structural abnormalities. In the current patients, MRI showed space-occupying lesions as well as flattening of the median nerve at the hamate level, palmar bowing of the flexor retinaculum, and increased signal intensity of the median nerve, all of which are typical MRI findings of CTS [7]. MRI also revealed a high signal intensity pattern in thenar muscles consistent with acute denervation in Case 2.

CTS presents bilaterally in 59% to 87% of patients [1, 2, 8], and approximately half of patients with unilateral symptoms were reported to have positive neurophysiological test results in the asymptomatic, contralateral hand [3, 8]. Follow-up of these patients with unilateral symptoms but bilateral neurophysiological impairment showed that contralateral symptoms developed in most cases and it is assumed that in some cases bilaterality may be time dependent. Hence, a patient with longstanding unilateral symptoms with severe neurophysiologic impairment and no neurophysiologic findings in the contralateral hand should alert the physician about the probable secondary nature of the disorder [1]. The incidence of space-occupying lesions in unilateral CTS is also higher than that of bilateral CTS. Nakamichi and Tachibana reported an increased incidence of space-occupying lesions in unilateral versus bilateral CTS and concluded that a space-occupying lesion should be suspected when the condition is unilateral and the etiology is unclear [4]. The reported patient was evaluated in this regard and MRI studies revealed an intratunnel lesion compressing the median nerve and the flexor tendons.

Although ganglia are common soft tissue tumors in the wrist, compression of peripheral nerves by ganglia is unusual and only a few cases have been reported in the literature [9, 10]. Space occupying lesions in the carpal tunnel can be missed and symptoms may not improve after carpal tunnel release [10, 11]. If a space-occupying lesion is detected, MRI can also delineate the relationship between the lesion and adjacent structures allowing the surgeon to plan the operation.
